# Constellations of Family Qualities and Links with Psychological and Behavioral Health in Adolescence and Young Adulthood

**DOI:** 10.1007/s10826-025-03154-4

**Published:** 2025-09-08

**Authors:** Anna K. Hochgraf, Mikayla R. Barry, Stephanie T. Lanza, Marlena Jacobsen, Dianne Neumark-Sztainer

**Affiliations:** 1 Division of Epidemiology and Community Health, School of Public Health, The University of Minnesota, Minneapolis, MN USA; 2 Department of Biobehavioral Health, Pennsylvania State University, University Park, PA USA

**Keywords:** Family relations, Adolescent, Young adult, Mental health, Latent class analysis

## Abstract

Positive family qualities, including low parent pressure to control weight, high physical activity support, frequent family meals, family connectedness, healthy family functioning, and parental monitoring, may promote youth psychological and behavioral health. We aimed to identify naturally occurring patterns of family qualities during adolescence and examine links with body satisfaction, self-esteem, depressive symptoms, disordered eating, and substance use during adolescence and young adulthood. Our goal was to inform family-centered interventions to prevent adverse health outcomes impacting youth. Data were from a longitudinal study of 1568 youth (53% female; 20% Asian, 29% Black, 17% Latinx, 19% White), that spanned adolescence (*M* age = 14.4 years) to young adulthood (*M* age = 22.2 years). Results from latent class analysis indicated that 8% of families were thriving, with low probability of parent pressure to control weight and high probabilities of physical activity support, frequent family meals, family connectedness, healthy family functioning, and parental monitoring. Other classes were distinguished by weight-specific risk (23% of families), broad risk (34% of families), disengagement (18% of families), and high risk (16% of families). Youth in thriving families reported better psychological and behavioral health than their peers concurrently in adolescence and longitudinally in young adulthood; yet this pattern of family qualities was rare. Family-centered interventions that target parent pressure to control weight, physical activity support, family meals, family connectedness, family functioning, and parental monitoring may help prevent multiple psychological and behavioral health problems. Heterogeneity in family qualities suggests that family-centered interventions could be tailored based on family strengths.

## Highlights

- We identified five naturally occurring patterns of family risk and protective factors for youth mental and behavioral health.
- Certain combinations of family qualities appear more beneficial for youth mental and behavioral health than others.
- Mitigating parent pressure to control weight may help prevent multiple health concerns in adolescence and young adulthood.

Psychological and behavioral disorders are highly prevalent, and most develop within the first two decades of life (Kessler & Wang, 2008). Adolescence is a period of opportunity for promoting positive psychological and behavioral health outcomes by bolstering protective factors and mitigating risk factors. Family Systems Theory (Cox & Paley, 1997; Minuchin, 1974) and prior research suggests that certain family qualities may confer risk for or protection against psychological and behavioral health problems that tend to develop during adolescence, including body dissatisfaction, low self-esteem, depressive symptoms, disordered eating, and substance use. Although development is shaped by a multitude of risk and protective factors (Bronfenbrenner & Morris, 2006), most extant research has focused on the role of one or two risk or protective factors at a time. The current study builds upon prior research by elucidating naturally occurring patterns of family qualities and examining how family qualities work in tandem to shape risk for body dissatisfaction, low self-esteem, depressive symptoms, disordered eating, and substance use during adolescence and young adulthood. Based on extant literature, we focused on six family qualities: parent pressure to control weight, family physical activity support, family meals, family connectedness, family functioning, and parental monitoring. The purpose of this study was to identify targets for family-centered interventions with the potential to help prevent a broad range of common psychological and behavioral health concerns; opportunities to streamline interventions based on family strengths; and to inform theory development.

Psychological and behavioral health concerns rank among the primary causes of illness and disability for adolescents (World Health Organization, 2024). Body dissatisfaction, low self-esteem, depressive symptoms, disordered eating, and substance use typically emerge and escalate during adolescence and can have detrimental effects on youth wellbeing and quality of life (Birkeland et al., 2012; Chen & Jacobson, 2012; McLean et al., 2022; Measelle et al., 2006; Otto et al., 2017; Schubert et al., 2017; Wang et al., 2019). There is evidence that for many youth, these psychological and behavioral health concerns persist or increase as youth transition into adulthood (Birkeland et al., 2012; Chen & Jacobson, 2012; Neumark-Sztainer et al., 2018; Schubert et al., 2017; Wang et al., 2019). Therefore, research on risk and protective processes during adolescence that set the stage for individuals’ long-term experiences of psychological and behavioral health is critical for advancing theory and prevention efforts.

Families have long been recognized for their role in fostering healthy development and protecting against development of adverse psychological and behavioral health outcomes among youth (Kumpfer & Alvarado, 2003; Minuchin, 1974; Parke & Buriel, 2006). Family Systems Theory and ecological models of development suggest that families are a primary context for youth development and are influential socialization agents (Minuchin, 1974; Parke & Buriel, 2006). That is, families have a central role in the process through which youth develop and conform to attitudes, skills, and behaviors that are normative and desirable within their broader social environment (e.g., societal beauty ideals and weight-related attitudes, attitudes toward substance use; Parke & Buriel, 2006). Family Systems Theory suggests that due to the interdependence of family systems, an individual’s development can only be understood within the context of their family system, and their psychological and behavioral adjustment is a reflection of family dynamics (Cox & Paley, 1997). Perhaps unsurprisingly, effective parenting has been described as the “most powerful” means of preventing behavioral health problems among adolescents (Kumpfer & Alvarado, 2003).

A growing body of research suggests that certain family qualities, namely parent pressure to control weight, family physical activity support, family meals, family connectedness, family functioning, and parental monitoring, may confer risk or protection for the development of psychological and behavioral health problems. Cross-sectional and longitudinal research has shown that parent pressure to control weight (i.e., encouragement to diet, weight talk, parent dieting behavior) predicts higher body dissatisfaction during adolescence and young adulthood (Neumark-Sztainer et al., 2010; Rodgers et al., 2021; Wang et al., 2019), as well as lower self-esteem, higher depressive symptoms, and higher risk for disordered eating in young adulthood (Berge et al., 2019). Findings from a short-term longitudinal study of adolescents suggest that family physical activity support may be protective against body dissatisfaction (Savage et al., 2009). A systematic review revealed that family meals are associated with lower body dissatisfaction, higher self-esteem, lower depressive symptoms, and lower risk for disordered eating and substance use (Harrison et al., 2015). However, there is stronger evidence for links between family meals and behavioral health outcomes than for psychological health outcomes (Harrison et al., 2015; Langdon-Daly & Serpell, 2017). Family connectedness (i.e., closeness and warmth between family members), has been longitudinally linked with lower body dissatisfaction (Boutelle et al., 2009; Crespo et al., 2010; Krug et al., 2016; May et al., 2006; Wang et al., 2019), higher self-esteem (Amato & Fowler, 2002; Boutelle et al., 2009; Hochgraf et al., 2018), lower depressive symptoms (Boutelle et al., 2009), and lower risk for disordered eating (Krug et al., 2016) and substance use (Mak & Iacovou, 2019; Van Ryzin et al., 2012). A systematic review indicated that healthy family functioning, which describes the way that family members interact, solve problems, communicate, and maintain relationships, is generally associated with better psychological health during adolescence (Scully et al., 2020). Healthy family functioning has also been cross-sectionally linked with lower risk of disordered eating (Berge et al., 2014) and longitudinally linked with lower risk of substance use during adolescence (Cordova et al., 2014). Longitudinal studies indicate that parental monitoring, which refers to parents’ tracking and awareness of their children’s whereabouts, activities, and companions, is associated with lower body dissatisfaction (Krug et al., 2016; May et al., 2006), higher self-esteem (Amato & Fowler, 2002), and lower risk for disordered eating (Krug et al., 2016) and substance use (Amato & Fowler, 2002; Van Ryzin et al., 2012). There is also evidence from evaluations of family interventions that cultivating positive family relationships (e.g., family connectedness, functioning) and effective parenting practices (e.g., parental monitoring) can mitigate psychological and behavioral health concerns among adolescents, including depressive symptoms, disordered eating, and substance use (Kumpfer & Alvarado, 2003; Kumpfer & Magalhães, 2018; Van Ryzin & Nowicka, 2013).

Studies of family risk and protective factors for the development of psychological and behavioral health outcomes often only focus on one or two family qualities, and yet from a developmental perspective, risk and protective factors do not exist in isolation. Rather, individuals experience a multitude of risk and protective factors that together shape developmental outcomes (Bronfenbrenner & Morris, 2006). For example, LoBraico et al. (2020) identified four constellations of family risk and protective factors: high functioning (low risk; low family conflict, positive family climate, high parental involvement, effective discipline, adolescent positive engagement, high parental monitoring), disengaged, permissive, and coercive (high risk). Adolescents in high risk families were at highest risk for antisocial behavior, and adolescents in low risk families were at lowest risk for antisocial behavior two years later (LoBraico et al., 2020). An essential step to advance knowledge and family-focused prevention efforts is to examine naturally occurring patterns or constellations of family risk and protective factors, and how these patterns are associated with psychological and behavioral health outcomes during adolescence and young adulthood. This novel research will contribute to the development of a comprehensive theory of the roles of families in youth development of psychological and behavioral health outcomes, address which sets of factors are associated with more desirable short- and long-term health outcomes, and inform family-centered prevention programming. Existing family-centered interventions that aim to prevent psychological or behavioral adjustment problems among youth rarely address body dissatisfaction or disordered eating behavior (Hart et al., 2014; *Program Search*, 2024). However, given that there is some overlap between family risk and protective factors for body dissatisfaction, disordered eating, self-esteem, depressive symptoms, and substance use, it may be possible to utilize family-centered programs to prevent body dissatisfaction and disordered eating in addition to more commonly addressed outcomes. This study may reveal intervention targets that efficiently prevent a broad range of psychological and behavioral health outcomes. Findings may also suggest opportunities for streamlined prevention strategies. For example, there may be subgroups in the population that would benefit from support on a limited set of family factors, which could reduce burden on families among other intervention costs.

## The Current Study

The current study addresses an important gap in knowledge about how family qualities work together to shape short- and long-term psychological and behavioral health outcomes during adolescence and young adulthood. The information gleaned from this study may have important implications for family-centered prevention strategies and theory regarding how families contribute to youth development. Guided by ecological models of development and Family Systems Theory, the current study builds upon extant literature by characterizing naturally occurring patterns of family risk and protective factors and testing concurrent and prospective associations with psychological and behavioral health outcomes within a community sample of youth from diverse socioeconomic and racial/ethnic backgrounds. The diversity within our sample is important given the historic lack of attention to youth from diverse backgrounds in developmental research, as well as inequities in access to mental health services experienced by youth from socioeconomically disadvantaged families and youth of color (Garcia Coll, 2015; Gudiño et al., 2009; Halbeisen et al., 2022). We hypothesized that several constellations of family qualities would emerge, including a low risk, or “Thriving” pattern, characterized by low parent pressure to control weight, high physical activity support, frequent family meals, high family connectedness, high family functioning, and high parental monitoring, as well as a “High Risk” pattern characterized by a paucity of protective factors. We expected to find Thriving and High Risk constellations because positive family qualities are often moderately correlated with each other and a previous study identified low and high risk family types (LoBraico et al., 2020). Due to the large number of potential combinations of family qualities, additional constellations of family qualities were exploratory. Based on extant research that points to low parent pressure to control weight, high physical activity support, frequent family meals, high family connectedness, high family functioning, and high parental monitoring as protective factors (Amato & Fowler, 2002; Berge et al., 2019; Boutelle et al., 2009; Harrison et al., 2015; Krug et al., 2016; Savage et al., 2009; Scully et al., 2020; Van Ryzin et al., 2012; Wang et al., 2019), we expected that youth in Thriving families during adolescence would have higher levels of body satisfaction and self-esteem, and lower levels of depressive symptoms, disordered eating, and substance use during adolescence and young adulthood relative to youth in other family types.

## Method

### Participants and Procedures

Adolescents were recruited to participate in Project Eating and Activity over Time (EAT), an epidemiological, longitudinal study of eating and weight-related health. Participants were recruited from 20 middle and high schools in the midwestern U.S. in the 2009–2010 school year (Time 1). Parents provided informed consent and youth provided informed assent for study participation at Time 1. At Time 2, youth reviewed the consent form. Completion of the Time 2 survey implied written consent. At Time 1, trained staff administered surveys to adolescents (*M* age = 14.4 years, *SD* = 0.05) in select health, physical education, and science classes. Participants were invited to complete follow-up surveys via mail or online in 2017–2018 (Time 2), when they were young adults (*M* age = 22.2 years, *SD* = 0.05). The analytic sample consisted of 1568 youth who completed surveys at both Times 1 and 2. Inverse probability weights were created to account for attrition between Times 1 and 2 (Seaman & White, 2013) and were applied to all analyses. These weights were designed to minimize bias due to missing data from attrition and ensure that the population represented was the same as in Time 1. The variables used to calculate inverse probability weights were: age, sex, race, ethnicity, U.S. born, socioeconomic status, dieting behavior, and body mass index. For more details on the inverse probability weights created for this study, see (Hooper et al. 2021). The sample was socioeconomically and racially/ethnically diverse; most participants were youth of color and 32% of adolescents reported that their family received a form of public assistance, such as food support. Slightly more than half of adolescents were female (see Table 1 for additional demographic information on the analytic sample). All study procedures were approved by the university’s institutional review board (submission ID: CR00013728, EAT 2010-2018: A Longitudinal Multi-contextual Study of Weight-Related Problems).

**Table 1:** Weighted Means or Frequencies of Demographic Characteristics and Study Variables

Variable	Frequency (%) or Mean (*SE*)
Age in Years at Time 1	14.43 (0.05)
Age in Years at Time 2	22.15 (0.05)
Socioeconomic Status Time 1	2.27 (0.03)
Gender	
Female	835 (53.24%)
Male	733 (46.76%)
Race	
White	294 (18.83%)
Black	454 (29.07%)
Latinx	265 (16.98%)
Asian	311 (19.91%)
Native American	58 (3.71%)
Native Hawaiian or Pacific Islander	9 (0.57%)
Other	171 (10.95%)
Mother Pressure to Control Weight Time 1	
No	286 (18.26%)
Yes	1228 (78.31%)
Father Pressure to Control Weight Time 1	
No	532 (33.91%)
Yes	779 (49.69%)
Family Physical Activity Support Time 1	
None	328 (20.94%)
Any	1223 (78.00%)
Frequency of Family Meals Time 1	
Infrequent ( < 5 times per week)	1095 (69.87%)
Frequent ( ≥ 5 times per week)	455 (29.04%)
Family Connectedness Time 1	
Low	253 (16.15%)
Moderate	521 (33.25%)
High	771 (49.16%)
Family Functioning Time 1	
Low	699 (44.57%)
High	855 (54.51%)
Parental Monitoring Time 1	
Low	864 (55.08%)
High	680 (43.35%)
Body Satisfaction Time 1	6.93 (1.61)
Body Satisfaction Time 2	14.41 (2.92)
Self-Esteem Time 1	18.17 (4.04)
Self-Esteem Time 2	24.39 (3.78)
Depressive Symptoms Time 1	19.16 (4.53)
Depressive Symptoms Time 2	24.74 (4.00)
Disordered Eating Time 1	
None	877 (55.92%)
Any	681 (43.41%)
Disordered Eating Time 2	
None	733 (46.76%)
Any	804 (51.28%)
Cigarette Use Time 1	
None	1370 (87.39%)
Any	160 (10.24%)
Cigarette Use Time 2	
None	1138 (72.60%)
Any	382 (24.38%)
Vaping Time 2	
None	1310 (83.55%)
Any	205 (13.10%)
Alcohol Use Time 1	
None	1178 (75.12%)
Any	355 (22.65%)
Binge Drinking Time 2	
None	951 (60.67%)
Any	574 (36.58%)

Percentages may not add up to 100 due to missing data. Unstandardized means for the psychological health outcomes are presented for descriptive purposes; standardized scores were used in analyses.

### Measures

Measures were selected based on theory, a literature review, and to allow for comparison with population-based studies. The Project EAT research team gathered input on the measures via focus groups conducted with diverse youth and via review by content experts. All measures used in this study were found to be psychometrically sound (Neumark-Sztainer et al., 2002).

#### Family qualities

Family qualities were measured at Time 1. To facilitate latent class analysis (LCA), we created a binary or categorical indicator for each family quality based on the distribution of scores, substantive interpretation of the score, and precedent set in the literature. Parent pressure to control weight was assessed with four items (e.g., “My mother/father encourages me to diet to control my weight”) that captured parents’ dieting behavior, comments about their own and others’ weight, and encouragement to diet (Neumark-Sztainer et al., 2003). Responses ranged from 1 = *not at all* to 4 = *very much* and Cronbach’s α = 0.65 for mothers and 0.72 for fathers. Scale scores were calculated for each participant by taking the mean of the four items and then transforming this into a binary variable. Based on the interpretation of the original scale, mean scores equal to one were coded as “0” to represent absence of pressure to control weight, and mean scores above one were coded as “1” to represent presence of pressure to control weight. Research suggests that mothers may be more likely to engage in pressure to control weight than fathers (Berge et al., 2019). Therefore, scores were calculated separately for mothers and fathers to allow constellations of family qualities to emerge in which mothers and fathers exhibited differential pressure to control weight. Family support for physical activity was measured with two items (e.g., “My family supports me in being physically active,” 1 = *strongly disagree* to 4 = *strongly agree*; Pearson’s *r* = 0.73). One item was developed for this survey and the other was adapted from prior research (Davison, 2004). A binary variable was created from the mean of the two items (0 = *low physical activity support (mean score* ≤ *2)*, 1 = *high physical activity support (mean score* > *2)*). Adolescents reported the frequency of family meals in the past seven days with the item, “During the past seven days, how many times did all, or most, of your family living in your house eat a meal together?” with response options ranging from 1 = *never* to 6 = *more than 7 times* (Neumark-Sztainer et al., 2003, 2004). Guided by a systematic review of family meals (Harrison et al., 2015), we created a binary variable representing frequent family meals (0 = *less than five meals per week*, 1 = *five or more meals per week*). Family connectedness was assessed with four items (e.g., “How much do you feel your mother/father cares about you?”) adapted from the Minnesota Adolescent Health Survey (Blum et al., 1989; Neumark-Sztainer et al., 2003, 2004; Resnick et al., 1993). Responses ranged from 1 = *not at all* to 5 = *very much* (Cronbach’s α = 0.67). Items were mean-scored and transformed into a three-level variable based on the interpretation of the original scale (0 = *low (1 ≤ mean score* < *3)*, 1 = *moderate (3 ≤ mean score* < *4)*, 3 = *high connectedness (4 ≤ mean score* ≤ *5)*). Family functioning was assessed with six items (e.g., “Family members are accepted for who they are”) from the General Functioning subscale of the Family Assessment Device (Epstein et al., 1983). Responses ranged from 1 = *strongly disagree* to 4 = *strongly agree*, and internal consistency was acceptable (Cronbach’s α = 0.70). We created a binary variable (0 = *unhealthy family functioning*, 1 = *healthy family functioning*) based on established clinical cutoffs for this scale (Miller et al., 1985). As we used a reverse coded version of family functioning in this study so that higher scores represented healthier functioning, scale scores greater than or equal to three were considered healthy family functioning and assigned a value of “1”. Parental monitoring was assessed with a six item scale (e.g., “How much does your mother/father really know where you are most afternoons after school?”) that captured parents’ awareness of their adolescents’ whereabouts and activities (Brown et al., 1993). Responses ranged from 1 = *doesn’t know* to 3 = *knows a lot* and Cronbach’s α = 0.81. Items were mean-scored and transformed into a binary variable based on the interpretation of the original scale (0 = *low monitoring (mean score* < *2.5)*, 1 = *high monitoring (mean score* ≥ *2.5)*).

#### Psychological health

Psychological health was assessed at Times 1 and 2. Body satisfaction was calculated as the mean of 10 items (e.g., “How satisfied are you with your stomach?”) that assessed satisfaction with different aspects of the body (Neumark-Sztainer et al., 2004; Pingitore et al., 1997). Responses ranged from 1 = *very dissatisfied* to 5 = *very satisfied* and Cronbach’s α = 0.93 at both timepoints. Depressive symptoms were calculated as the mean of four items (e.g., “During the past 12 months, how often have you been bothered or troubled by feeling unhappy, sad, or depressed?”; Kandel & Davies, 1982). Responses ranged from 1 = *not at all* to 3 = *very much* and Cronbach’s α = 0.74 at Time 1 and α = 0.84 at Time 2. Self-esteem was calculated as the mean of six items from the Rosenberg Self-Esteem Scale (Rosenberg, 1965). Participants were asked how strongly they agreed with each of the six statements (e.g., “I feel that I have a number of good qualities”) on a scale from 1 = *strongly disagree* to 4 = *strongly agree*. Cronbach’s α = 0.79 at Time 1 and α = 0.81 at Time 2. Body satisfaction, depressive symptoms, and self-esteem scores were standardized within wave to facilitate comparison across outcomes. Higher scores represented higher body satisfaction, depressive symptoms, and self-esteem.

#### Behavioral health

Behavioral health was assessed at Times 1 and 2. Participants reported whether they engaged in disordered eating behavior (i.e., fasting, eating very little food, taking diet pills, self-induced vomiting, use of laxatives, use of diuretics, use of a food substitute, skipping meals, smoking more cigarettes) during the past year with nine *no/yes* items (Neumark-Sztainer et al., 2003). We created a binary variable to indicate engagement in any disordered eating behavior (0 = *none*, 1 = *any*). Substance use measures were adapted from the Minnesota Adolescent Health Survey (Blum et al., 1989). Participants reported how often they engaged in cigarette and alcohol use respectively during the past year (1 = *never* to 5 = *daily*). Due to the infrequency of substance use at Time 1, we used these two items to create binary indicators for each substance (0 = *none*, 1 = *any*). As infrequent and low intensity alcohol use is normative and not necessarily problematic during young adulthood (National Institute on Alcohol Abuse and Alcoholism, 2023; Patrick et al., 2023), we indexed alcohol use at Time 2 with a binary variable that indicated whether participants engaged in binge drinking (i.e., consumed five or more alcoholic drinks in one sitting) during the past two weeks (0 = *did not binge drink*, 1 = *at least once*). At Time 2, participants also reported frequency of nicotine vaping during the past year, which we coded as 0 = *none*, 1 = *any* due to infrequent use in this sample.

#### Covariates

Age in years, gender, and socioeconomic status (SES) were assessed at Time 1. Age was centered on the weighted mean age of the sample for ease of interpretation. SES was based on parent employment status, household income, and parent educational attainment. Categories of SES ranged from 1 - 5 and were determined based on results from a prior factor analysis that yielded a single factor with approximately equal weights across employment, income, and education (Neumark-Sztainer et al., 2008). Cutoffs for SES categories were set in sparse regions of the distribution of the factor score to minimize misclassification of individuals. Higher values represent higher SES.

### Analyses

As a descriptive step, we calculated weighted frequencies and means for demographic and family characteristics as well as psychological and behavioral health outcomes. We used LCA with distal outcomes (Lanza et al., 2013; Lanza & Cooper, 2016) to address our research questions. LCA is a type of finite mixture model that enables identification of subgroups in the population based on categorical indicators. We chose LCA instead of latent profile analysis (LPA; uses continuous indicators) for two reasons. First, several of our variables of interest were not normally distributed and could not be transformed to meet this important assumption of LPA. Second, LCA is potentially more clinically useful than LPA, as it enables estimation of the probability of the presence (or absence) of risk and protective factors based on class membership. LCA with distal outcomes enables prediction of observed outcomes of interest based on class membership (Lanza et al., 2013). In this case, we used LCA to identify groups of families that were similar in their patterns of risk and protective factors and then assessed concurrent and longitudinal associations with youth psychological and behavioral health outcomes. First, we completed model selection by comparing fit indices and interpretability of models that differed in number of classes, ranging from 1 to 7 classes. Next, we examined associations between class membership and psychological and behavioral health outcomes by adding these outcomes to the model one at a time. We conducted planned comparisons between the lowest risk class and the other classes to test our hypothesis that youth in the lowest risk class during adolescence would fare better than youth in other classes in terms of psychological and behavioral health during adolescence and young adulthood. We used the Bolck-Croon-Hagenaars (BCH) method, a model-based approach to estimating associations between latent class membership and outcomes that corrects for classification error inherent in latent variables (Bakk & Vermunt, 2016; Bolck et al., 2004). This 3-step approach uses parameter estimates from a baseline measurement model to calculate BCH weights that are subsequently applied when modeling outcomes as a function of latent class analysis (Asparouhov & Muthen, 2021). Linear regression was used for continuous outcomes (i.e., the psychological health outcomes) and logistic regression was used for binary outcomes (i.e., the behavioral health outcomes). All models were adjusted for age, gender, and SES. As a supplementary, more conservative test of class differences in long-term health outcomes, we also included health outcomes at Time 1 as covariates in models estimating health outcomes in young adulthood (Time 2). This supplementary analysis indicates whether class differences in psychological and behavioral health outcomes remain after adjusting for prior levels of the outcome.

### Transparency and Openness

We reported how we determined the sample for this study and the rationale for data exclusion. We also reported all measures used in the analyses for this study. The data, syntax, and materials may be made available upon reasonable request to the Project EAT principal investigator. This study was not preregistered. Descriptive statistics were calculated using SAS software version 9.4 (SAS Institute, 2016) and LCA and regression analyses were conducted in Mplus version 8.9 (Muthen & Muthen, 2017) using syntax adapted from a prior study (Bray et al., 2023) to accommodate weights and covariates.

## Results

### Descriptive Statistics

Descriptive statistics for the analytic sample are presented in Table 1. Approximately three quarters (78%) of adolescents reported that they experienced pressure to control weight from their mothers, whereas half (50%) of adolescents reported pressure to control weight from their fathers. Three quarters (78%) of adolescents reported physical activity support from their families, and less than one third (29%) of adolescents reported frequent family meals. More than three quarters (82%) of adolescents reported moderate or high family connectedness, and slightly more than half (55%) of adolescents reported healthy family functioning. Less than half (43%) of adolescents reported high parental monitoring.

### Constellations of Family Qualities

A five-class model was selected based on fit indices and interpretability of the classes (Supplementary Table 1). Response probabilities for each of the latent classes are presented in Table 2. Approximately 8% of adolescents were sorted into a low risk or “Thriving” class, characterized by low probability of parent pressure to control weight, and high probabilities of family physical activity support, frequent family meals, family connectedness, healthy family functioning, and high parental monitoring. Approximately 23% of adolescents were sorted into a “Weight-Specific Risk” class, which was characterized by high probability of pressure to control weight from parents, but otherwise was comparable to the thriving class. The largest class was the “Broad Risk” class, which described 34% of adolescents and was distinguished by high probabilities of pressure to control weight from parents and moderate risk across the remaining indicators. A “Disengaged” class described 18% of adolescents and was distinguished by relatively low probabilities of pressure to control weight from parents, frequent family meals, and parental monitoring, and high probability of healthy family functioning. Approximately 16% of adolescents were sorted into a “High Risk” class, which was marked by high probability of parent pressure to control weight and low probabilities of physical activity support, frequent family meals, family connectedness, healthy family functioning, and parental monitoring.

**Table 2:** Probability of Endorsing Each Family Quality Within Each Latent Class

	Latent Class				
	Thriving 8% *n* = 129	Weight-Specific Risk 23% *n* = 363	Broad Risk 34% *n* = 534	Disengaged 18% *n* = 286	High Risk 16% *n* = 253
*Item Response Probabilities:*					
Mother Pressure to Control Weight					
No	1.00	0.00	0.03	0.40	0.16
Yes	0.00	1.00	0.98	0.60	0.85
Father Pressure to Control Weight					
No	0.70	0.27	0.10	0.90	0.54
Yes	0.30	0.73	0.90	0.10	0.46
Family Physical Activity Support					
None	0.07	0.01	0.20	0.23	0.59
Any	0.93	0.99	0.80	0.77	0.41
Frequency of Family Meals					
Infrequent ( < 5 times per week)	0.44	0.48	0.82	0.75	0.89
Frequent ( ≥ 5 times per week)	0.56	0.53	0.18	0.25	0.11
Family Connectedness					
Low	0.00	0.01	0.09	0.15	0.64
Moderate	0.08	0.04	0.55	0.41	0.37
High	0.92	0.95	0.37	0.44	0.00
Family Functioning					
Low	0.22	0.05	0.63	0.27	0.96
High	0.78	0.95	0.37	0.73	0.04
Parental Monitoring					
Low	0.10	0.20	0.64	0.78	0.88
High	0.90	0.80	0.36	0.22	0.13

Family qualities were assessed at Time 1 (adolescence).

### Class Differences in Psychological Health During Adolescence and Young Adulthood

**Fig. 1:**
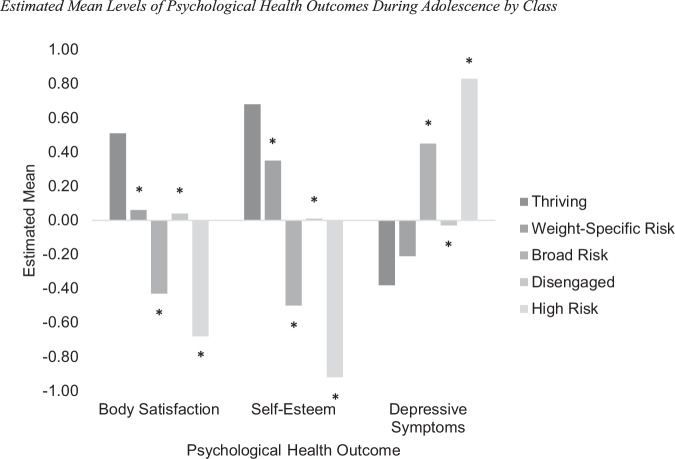
Estimated Mean Levels of Psychological Health Outcomes During Adolescence by Class. Asterisks denote a statistically significant difference (*p* < .05) in the outcome relative to the Thriving class. Models were adjusted for age, gender, and socioeconomic status. The pattern of estimated means across classes was similar in young adulthood

As shown in Fig. 1, the Thriving class had mean levels of body satisfaction, self-esteem, and depressive symptoms during adolescence that suggest positive psychological health. Class differences in psychological health are shown in Table 3. As family qualities were assessed during adolescence, class differences in psychological health during adolescence are concurrent associations, and class differences during young adulthood are longitudinal associations. Relative to the Thriving class, all other classes had significantly lower mean levels of body satisfaction and self-esteem and significantly higher mean levels of depressive symptoms during adolescence. The only exception to this was for the Weight-Specific Risk class, which did not significantly differ from the Thriving class in depressive symptoms during adolescence. In young adulthood, the Broad Risk and High Risk classes still exhibited significantly lower levels of body satisfaction and self-esteem and significantly higher levels of depressive symptoms relative to the Thriving class. The Disengaged class also had significantly lower levels of self-esteem and higher depressive symptoms relative to the Thriving class. The supplementary analyses (Supplementary Table 2) revealed that after controlling for health outcomes in adolescence, youth in the High Risk class had significantly lower levels of self-esteem in young adulthood relative to the Thriving class. Youth in the Broad Risk, Disengaged, and High Risk classes also had significantly higher levels of depressive symptoms during young adulthood relative to the Thriving class.

**Table 3:** Concurrent and Longitudinal Class Differences in Psychological Health During Adolescence and Young Adulthood

Psychological Health Outcome	Thriving		Weight-Specific Risk		Broad Risk		Disengaged		High Risk	
Adolescence (T1)	*M*	*β* 95% CI	*M*	*β* 95% CI	*M*	*β* 95% CI	*M*	*β* 95% CI	*M*	*β* 95% CI
Body Satisfaction	0.51	*ref*	0.06	**−0.45**	−0.43	**−0.94**	0.04	**−0.47**	−0.68	**−1.19**
				−0.69, −0.22		−1.17, −0.93		−0.77, −0.18		−1.45, −0.93
Self-Esteem	0.68	*ref*	0.35	**−0.33**	−0.50	**−1.18**	0.01	**−0.68**	−0.92	**−1.60**
				−0.55, −0.12		−1.40, −0.97		−0.95, −0.40		−1.84, −1.36
Depressive Symptoms	−0.38	*ref*	−0.21	0.17	0.45	**0.83**	−0.03	**0.35**	0.83	**1.21**
				−0.03, 0.36		0.63, 1.04		0.10, 0.61		0.96, 1.46
Young Adulthood (T2)										
Body Satisfaction	0.04	*ref*	−0.16	−0.20	−0.47	**−0.51**	−0.11	−0.15	−0.57	**−0.61**
				−0.42, 0.03		−0.74, −0.29		−0.44, 0.14		−0.87, −0.35
Self-Esteem	0.32	*ref*	0.17	−0.16	−0.17	**−0.49**	−0.08	**−0.40**	−0.40	**−0.72**
				−0.40, 0.09		−0.72, −0.25		−0.71, −0.10		−0.99, −0.45
Depressive Symptoms	−0.22	*ref*	−0.23	−0.01	0.25	**0.47**	0.15	**0.37**	0.29	**0.51**
				−0.23, 0.22		0.25, 0.70		0.08, 0.66		0.26, 0.77

Psychological health outcomes are standardized. Models were adjusted for age, gender, and socioeconomic status. Class differences are concurrent in adolescence and longitudinal in young adulthood. Bolded coefficients are statistically significant at *p* < .05. The *n*’s used to estimate each model were as follows: T1 body satisfaction = 1527, T1 self-esteem = 1524, T1 depressive symptoms = 1522, T2 body satisfaction = 1515, T2 self-esteem = 1498, T2 depressive symptoms = 1497.

*M* estimated mean, *T1* Time 1, *T2* Time 2

### Class Differences in Behavioral Health in Adolescence and Young Adulthood

**Fig. 2:**
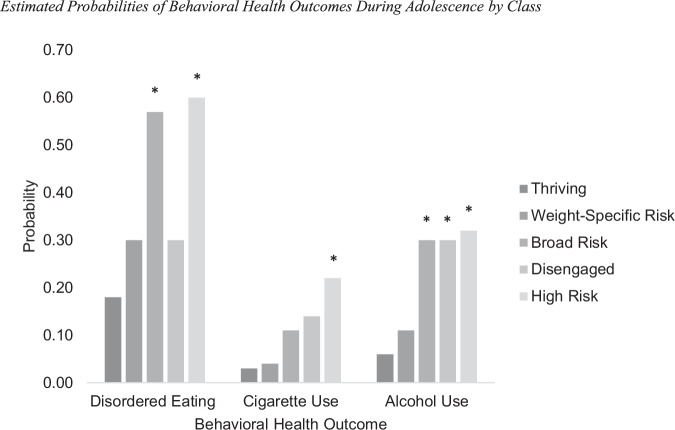
Estimated Probabilities of Behavioral Health Outcomes During Adolescence by Class. Asterisks denote a statistically significant difference (*p* < .05) in the outcome relative to the Thriving class. Models were adjusted for age, gender, and socioeconomic status. The pattern of probabilities for disordered eating and cigarette use across classes was similar in young adulthood.

As shown in Fig. 2, the Thriving class had low prevalences of disordered eating, cigarette use, and alcohol use during adolescence. Class differences in behavioral health outcomes are shown in Table 4. Class differences during adolescence are concurrent associations, and class differences during young adulthood are longitudinal associations. Compared to the adolescents in the Thriving class, adolescents in the Broad Risk class and High Risk class had significantly higher odds of disordered eating behavior. Adolescents in the High Risk class had significantly higher odds of cigarette use relative to the Thriving class. Adolescents in the Broad Risk class, Disengaged class, and High Risk class had significantly higher odds of alcohol use compared to adolescents in the Thriving class.

**Table 4:** Concurrent and Longitudinal Class Differences in Behavioral Health During Adolescence and Young Adulthood

Behavioral Health Outcome	Thriving		Weight-Specific Risk		Broad Risk		Disengaged		High Risk	
Adolescence (T1)	*P*	OR 95% CI	*P*	OR 95% CI	*P*	OR 95% CI	*P*	OR 95% CI	*P*	OR 95% CI
Disordered Eating										
Any	0.18	*Ref*	0.30	2.05	0.57	**6.16**	0.30	1.86	0.60	**5.94**
				0.69, 3.42		2.15, 10.17		0.37, 3.34		1.87, 10.02
Cigarette Use										
Any	0.03	*Ref*	0.04	1.43	0.11	3.95	0.14	5.24	0.22	**9.90**
				0.25, 8.13		0.78, 20.00		0.89, 30.30		1.98, 50.00
Alcohol Use										
Any	0.06	*Ref*	0.11	2.21	0.30	**6.94**	0.32	**6.99**	0.32	**7.63**
				0.66, 7.46		2.17, 22.22		1.96, 25.00		2.35, 25.00
Young Adulthood (T2)										
Disordered Eating										
Any	0.30	*Ref*	0.47	**2.24**	0.62	**3.82**	0.41	1.53	0.64	**3.69**
				1.29, 3.86		2.22, 6.58		0.78, 3.00		2.04, 6.67
Cigarette Use										
Any	0.12	*Ref*	0.20	1.75	0.23	2.18	0.26	**2.73**	0.39	**5.56**
				0.80, 3.86		0.99, 4.76		1.09, 6.80		2.48, 12.50
Vaping										
Any	0.08	*Ref*	0.10	1.10	0.18	**2.66**	0.14	2.07	0.12	1.88
				0.43, 2.82		1.11, 6.37		0.70, 6.06		0.71, 4.98
Alcohol Use										
Binge drinking at least once in the past two weeks	0.38	*Ref*	0.35	0.86 0.51, 1.45	0.40	1.06 0.64, 1.77	0.36	0.86 0.44, 1.64	0.42	1.25 0.71, 2.18

The Thriving class was the reference class for comparisons. Models were adjusted for age, gender, and socioeconomic status. Class differences are concurrent in adolescence and longitudinal in young adulthood. Bolded ORs are statistically significant at *p* < .05. The *n*’s used to estimate each model were as follows: T1 disordered eating = 1523, T1 cigarette use = 1509, T1 alcohol use = 1511, T2 disordered eating = 1502, T2 cigarette use = 1486, T2 vaping = 1481, T2 alcohol use = 1492.

*P* estimated probability, OR odds ratio, *T1* Time 1, *T2* Time 2

Youth who were in the Weight-Specific Risk class, Broad Risk class, and High Risk class during adolescence had significantly higher odds of disordered eating during young adulthood relative to youth who were in the Thriving class. This difference was most pronounced for youth in the Broad Risk and High Risk classes. Youth who were in the Disengaged class or High Risk class during adolescence had significantly higher odds of cigarette use during young adulthood compared to youth who were in the Thriving class. Youth who were in the Broad Risk class had significantly higher odds of vaping in young adulthood compared to youth in the Thriving class. There were no class differences in binge drinking during young adulthood. Results from the supplementary analyses (Supplementary Table 2) revealed that after controlling for behavioral health outcomes during adolescence, youth in the Weight-Specific Risk, Broad Risk, and High Risk classes had higher odds of disordered eating during young adulthood than youth in the Thriving class. Youth in the Broad Risk class had significantly higher odds of vaping during young adulthood relative to youth in the Thriving class.

## Discussion

Prior research has demonstrated that certain family qualities are individually associated with better health among adolescents and young adults. However, risk and protective factors do not occur in isolation; thus, the purpose of this study was to identify naturally occurring constellations of family qualities during adolescence and test associations with psychological and behavioral health outcomes during adolescence and young adulthood. The impetus for this research was to advance knowledge and theory about the role of family environments in fostering youth wellbeing and to inform development of family-centered prevention programming aimed at mitigating a broad range of psychological and behavioral health problems. We harnessed both cross-sectional and longitudinal data from a large and diverse community-based cohort of youth in the United States to address our research aims.

### Constellations of Family Qualities

Findings supported our hypothesis that multiple patterns of family qualities would emerge, including Thriving and High Risk patterns. LCA revealed five distinct patterns of family qualities: Thriving, Weight-Specific Risk, Disengaged, Broad Risk, and High Risk. Thriving families were characterized by low parent pressure to control weight, high physical activity support, frequent family meals, high family connectedness, high family functioning, and high parental monitoring. That is, Thriving family environments included a wealth of protective factors and few to no risk factors during adolescence. Thriving families were rare; only 8% of adolescents reported this constellation of family qualities. Weight-Specific Risk families were similar to Thriving families in most qualities but were distinguished by pressure to control weight from mothers and fathers. Broad Risk families were characterized by pressure to control weight from mothers and fathers, family physical activity support, infrequent family meals, unhealthy family functioning, moderate parent-adolescent connectedness, and low parental monitoring. This is concerning given that over one third of families were categorized as Broad Risk. The combination of family qualities observed among Disengaged families suggests that for the most part, these families had healthy family functioning and relatively low parent pressure to control weight, but that parents and adolescents were not connected and that parents had low awareness of their adolescents’ whereabouts or who their friends were. This suggests a lack of open, positive communication between parents and adolescents. As expected, the High Risk pattern was characterized by a paucity of protective factors, and approximately one in six adolescents were in High Risk families. Overall, these findings highlight heterogeneity in family qualities.

### Concurrent and Longitudinal Associations Between Family Qualities and Youth Wellbeing

Adolescents in Thriving families generally fared best with respect to psychological and behavioral health outcomes assessed concurrently in adolescence and eight years later in young adulthood. Adolescents in the High Risk class generally fared the worst across the mental and behavioral health outcomes examined in this study. These findings are consistent with theory (Cox & Paley, 1997; Minuchin, 1974; Parke & Buriel, 2006) and previous research showing that family risk and protective factors are linked to psychological and behavioral health outcomes among young people. For example, prior research indicates that parent pressure to control weight prospectively predicts body dissatisfaction (Wang et al., 2019), low self-esteem, depressive symptoms, and disordered eating (Berge et al., 2019). Frequent family meals are associated with lower body dissatisfaction, higher self-esteem, lower depressive symptoms, and lower risk for disordered eating and substance use (Harrison et al., 2015). Longitudinal studies suggest that family connectedness is associated with lower body dissatisfaction, higher self-esteem, and lower depressive symptoms (Boutelle et al., 2009), less disordered eating (Krug et al., 2016), and less substance use (Van Ryzin et al., 2012). Healthy family functioning appears to support youth psychological health (Scully et al., 2020), and longitudinal research suggests that parental monitoring is protective against body dissatisfaction and disordered eating (Krug et al., 2016), as well as self-esteem and substance use (Amato & Fowler, 2002). Our findings suggest that exposure to more family protective factors and fewer risk factors generally correlates with better health outcomes. This conclusion is consistent with a prior study that found that adolescents in families with multiple protective factors and limited risk factors were at lower risk for antisocial behavior compared to their peers (LoBraico et al., 2020). Our findings extend prior research by also demonstrating that family environments have short- and long-term implications for youth wellbeing.

Although the Thriving and Weight-Specific Risk classes differed only by parent pressure to control weight, adolescents in the Weight-Specific Risk class had significantly lower levels of body satisfaction and self-esteem during adolescence, as well as higher odds of disordered eating in young adulthood. This finding suggests that high parent pressure to control weight may have negative implications for body image, self-esteem, and disordered eating that are not fully buffered by coinciding protective factors. Given the many protective factors (e.g., physical activity support, frequent family meals, family connectedness) found in Weight-Specific Risk families, it is possible that these parents have positive but misinformed intentions for pressuring children to control their weight and are unaware of the potential harms of weight-focused pressure. Intervening on parent pressure to control weight might be an efficient strategy to help families, especially those with Weight-Specific Risk, lower adolescents’ risk for body image concerns, low self-esteem, and disordered eating. Future research exploring the efficacy of educating parents to refrain from pressure to control weight may be warranted.

One unexpected finding was that there was no significant difference in depressive symptoms between adolescents in the Thriving and Weight-Specific Risk classes. Although the mean level of depressive symptoms was higher within the Weight-Specific Risk class compared to the Thriving class, this difference was not statistically significant. One possible explanation for this finding is that parent pressure to control weight, the key difference between these two classes, may be more closely linked to adolescents’ body satisfaction and self-esteem than to their depressive symptoms. Adolescents’ perceptions of self-worth are often tied to how they feel about their bodies (Hochgraf et al., 2018). Indeed, perceived appearance has been proposed as one of the most important contributors to adolescents’ sense of self (Markey, 2010).

### Implications for Theory and Prevention

Findings from this study are aligned with Family Systems Theory and ecological models of development, which suggest that families are an important developmental context, and that youth development is shaped by a myriad of risk and protective factors (Bronfenbrenner & Morris, 2006; Minuchin, 1974; Parke & Buriel, 2006). An important next step is to integrate these findings with family and developmental theories and extant research to generate a more specific theory of the role of family environments and processes in youth development of psychological and behavioral health outcomes. In terms of implications for prevention, findings underscore the potential value of family-centered intervention programs for a range of psychological and behavioral health outcomes. Development is shaped via transactions between individuals and their environments, and as a proximal developmental context, families can provide some protection against stressors in adolescents’ broader socioecological context that may promote risk for adverse health outcomes. Our findings suggest that family interventions implemented during adolescence may confer benefits for youth development in adolescence and young adulthood. Intervention programming that deters families from pressuring adolescents to control their weight, fosters physical activity support, frequent family meals, high family connectedness, healthy family functioning, and parental monitoring, may promote body satisfaction and high self-esteem, and prevent depressive symptoms, disordered eating, and substance use. There are several evidence-based, family-centered prevention programs that target family relationships and parenting practices and have demonstrated benefits for adolescent health concerns, including substance use and depressive symptoms (Kumpfer & Alvarado, 2003; Kumpfer & Magalhães, 2018; Van Ryzin & Nowicka, 2013). It may be possible to adapt such programs to address a wider range of psychological and behavioral health concerns. Family-centered interventions aimed at preventing adverse psychological and behavioral health outcomes among adolescents generally do not address body satisfaction or disordered eating, and yet our findings suggest that this may be possible. In addition, the heterogeneity among families in this study suggests that it may be possible to tailor family prevention programs to families’ needs. For example, families could receive specific intervention components based on a screening or consultation about areas of strength and growth. This streamlined approach may be an efficient use of resources and ease the burden on families to participate in many sessions.

### Strengths and Limitations

This study has numerous strengths. We assessed a broad range of family qualities and mental and behavioral health outcomes within a large cohort of racially/ethnically and socioeconomically diverse adolescents. The longitudinal study design enabled us to test both concurrent and prospective associations between family qualities and youth health outcomes. Our longitudinal findings are particularly compelling given the large time interval between measurement occasions. Moreover, some of these findings remained statistically significant after controlling for the outcomes at Time 1, suggesting particularly robust longitudinal associations. Our use of LCA allowed us to examine naturally occurring constellations of family qualities. We also examined both risk and protective factors, which enabled us to identify classes comprised of unique combinations of risk and protective factors (e.g., the Weight-Specific Risk class).

This study also has some limitations. We measured family qualities at one time point during adolescence, but family qualities can be dynamic, meaning we may not have fully captured every participant’s family experience during adolescence. A future direction is to collect intensive data on family qualities during adolescence and investigate how families’ transitions between different constellations of family qualities are associated with long-term health outcomes. Due to the nature of self-reports, our measures may have been subject to recall bias. Some constructs of interest may have been underreported due to their potentially sensitive nature (e.g., alcohol use during adolescence). Additionally, we relied on adolescents’ reports for all constructs. However, a more comprehensive understanding of family qualities may be achieved by considering the perspectives of multiple family members. It is possible that loss to follow-up was not random; however, we used inverse probability weighting to minimize potential bias due to attrition. Finally, results from this observational study are correlational.

## Conclusions

Our findings reveal heterogeneity in family risk and protective factors for youth psychological and behavioral health outcomes. The combination of low parent pressure to control weight, family physical activity support, frequent family meals, high parent-adolescent connectedness, healthy family functioning, and high parental monitoring during adolescence was linked to better psychological and behavioral health in adolescence and young adulthood. Future research should aim to advance family-centered intervention efforts by seizing opportunities to prevent multiple health problems and by considering streamlined approaches to intervention delivery.

## Supplementary information

Constellations of Family Qualities_Supplementary Material
