# Supplementary material for: Constellations of Family Qualities and Links with Psychological and Behavioral Health in Adolescence and Young Adulthood
Source: J Child Fam Stud. 2025 Sep 8;34(10):2734–48. doi: 10.1007/s10826-025-03154-4 (PMC12470379; doi:10.1007/s10826-025-03154-4)
Supplement: Supplementary file 1 — Constellations of Family Qualities_Supplementary Material [file 10826_2025_3154_MOESM1_ESM.docx]

**Supplementary Material**

The following information supplements “Constellations of Family Qualities and Links with Psychological and Behavioral Health in Adolescence and Young Adulthood.”

**Supplementary Table 1**

Model Selection

| Number of  Latent Classes | Parameters  Estimated | *Degrees of*  *Freedom* | Chi Squared | AIC | BIC | Entropy |
| --- | --- | --- | --- | --- | --- | --- |
| 1 | 8 | 182 | 1073.11 | 14141.38 | 14184.22 | -- |
| 2 | 17 | 174 | 443.87 | 13358.50 | 13449.55 | 0.65 |
| 3 | 26 | 165 | 319.62 | 13235.18 | 13374.43 | 0.74 |
| 4 | 35 | 156 | 239.82 | 13162.53 | 13349.98 | 0.62 |
| **5** | **44** | **147** | **191.70** | **13126.02** | **13361.67** | **0.66** |
| 6 | 53 | 138 | 157.60 | 13105.49 | 13389.34 | 0.64 |
| 7 | 62 | 129 | 136.05 | 13099.27 | 13431.32 | 0.67 |

*Note.* AIC = Akaike’s Information Criterion, BIC = Bayesian Information Criterion.

**Supplementary Table 2**

Class Differences in Psychological and Behavioral Health Outcomes in Young Adulthood, Adjusted for Health Outcomes at Time 1

| Health Outcome | Thriving | | Weight-Specific Risk | | Broad Risk | | Disengaged | | High Risk | |
| --- | --- | --- | --- | --- | --- | --- | --- | --- | --- | --- |
|  | *M* | *β*  95% CI | *M* | *β*  95% CI | *M* | *β*  95% CI | *M* | *β*  95% CI | *M* | *β*  95% CI |
| Body Satisfaction | -0.12 | *ref* | -0.18 | -0.05 | -0.33 | -0.21 | -0.14 | -0.02 | -0.36 | -0.24 |
|  |  |  |  | -0.27, 0.16 |  | -0.44, 0.02 |  | -0.30, 0.26 |  | -0.50, 0.02 |
|  |  |  |  |  |  |  |  |  |  |  |
| Self-Esteem | 0.16 | *ref* | 0.07 | -0.08 | -0.04 | -0.20 | -0.07 | -0.23 | -0.18 | **-0.34** |
|  |  |  |  | -0.33, 0.16 |  | -0.45, 0.05 |  | -0.53, 0.07 |  | -0.63, -0.05 |
|  |  |  |  |  |  |  |  |  |  |  |
| Depressive Symptoms | -0.15 | *ref* | -0.18 | -0.03 | 0.16 | **0.31** | 0.15 | **0.30** | 0.15 | **0.29** |
|  |  |  |  | -0.26, 0.20 |  | 0.07, 0.54 |  | 0.01, 0.57 |  | 0.02, 0.57 |
|  |  |  |  |  |  |  |  |  |  |  |
|  | *P* | OR  95% CI | *P* | OR  95% CI | *P* | OR  95% CI | *P* | OR  95% CI | *P* | OR  95% CI |
| Disordered Eating |  |  |  |  |  |  |  |  |  |  |
| Any | 0.31 | *ref* | 0.47 | **1.99** | 0.62 | **2.88** | 0.41 | 1.36 | 0.64 | **2.78** |
|  |  |  |  | 1.14, 3.47 |  | 1.64, 5.05 |  | 0.68, 2.69 |  | 1.51, 5.08 |
| Cigarette Use |  |  |  |  |  |  |  |  |  |  |
| Any | 0.11 | *ref* | 0.19 | 1.75 | 0.23 | 2.11 | 0.26 | 2.54 | 0.39 | **4.78** |
|  |  |  |  | 0.75, 4.08 |  | 0.92, 4.88 |  | 0.95, 6.76 |  | 2.01, 11.36 |
| Vaping |  |  |  |  |  |  |  |  |  |  |
| Any | 0.08 | *ref* | 0.10 | 1.04 | 0.18 | **2.46** | 0.14 | 1.83 | 0.12 | 1.50 |
|  |  |  |  | 0.40, 2.69 |  | 1.02, 5.92 |  | 0.62, 5.43 |  | 0.55, 4.08 |
| Alcohol Use |  |  |  |  |  |  |  |  |  |  |
| Binge drinking at least once in the past two weeks | 0.38 | *ref* | 0.35 | 0.85 | 0.41 | 0.96 | 0.36 | 0.76 | 0.42 | 1.08 |
|  |  |  |  | 0.50, 1.44 |  | 0.57, 1.63 |  | 0.39, 1.47 |  | 0.61, 1.92 |

*Note.* *M* = estimated mean. *P* = probability. Psychological health outcomes are standardized. All models were adjusted for age, gender, and socioeconomic status at Time 1. In the model predicting vaping, cigarette use at Time 1 was included as a covariate in place of Time 1 vaping. In the model predicting binge drinking, any alcohol use at Time 1 was included as a covariate in place of Time 1 binge drinking. Bolded coefficients are statistically significant at *p*  < .05.
